# Zika Virus in the Male Reproductive Tract

**DOI:** 10.3390/v10040198

**Published:** 2018-04-16

**Authors:** Liesel Stassen, Charles W. Armitage, David J. van der Heide, Kenneth W. Beagley, Francesca D. Frentiu

**Affiliations:** Institute of Health and Biomedical Innovation, and School of Biomedical Sciences, Queensland University of Technology, Brisbane 4006, Queensland, Australia; liesel.stassen@qut.edu.au (L.S.); charles.armitage@qut.edu.au (C.W.A.); d.vanderheide@qut.edu.au (D.J.v.d.H.); k2.beagley@qut.edu.au (K.W.B.)

**Keywords:** flavivirus, arbovirus, Zika, sexual transmission, testis, prostate

## Abstract

Arthropod-borne viruses (arboviruses) are resurging across the globe. Zika virus (ZIKV) has caused significant concern in recent years because it can lead to congenital malformations in babies and Guillain-Barré syndrome in adults. Unlike other arboviruses, ZIKV can be sexually transmitted and may persist in the male reproductive tract. There is limited information regarding the impact of ZIKV on male reproductive health and fertility. Understanding the mechanisms that underlie persistent ZIKV infections in men is critical to developing effective vaccines and therapies. Mouse and macaque models have begun to unravel the pathogenesis of ZIKV infection in the male reproductive tract, with the testes and prostate gland implicated as potential reservoirs for persistent ZIKV infection. Here, we summarize current knowledge regarding the pathogenesis of ZIKV in the male reproductive tract, the development of animal models to study ZIKV infection at this site, and prospects for vaccines and therapeutics against persistent ZIKV infection.

## 1. Emergence of Zika Virus

Zika virus (ZIKV), a previously obscure and scientifically neglected virus, became a serious public health concern in 2015 due to an association with microcephaly (refer to Glossary) in Brazil [1]. ZIKV is a positive-sense, nonsegmented, enveloped, single-stranded RNA virus that belongs to the *flavivirus* genus within the *Flaviviridae* family [2,3]. The genus also includes other medically important flaviviruses such as dengue, yellow fever, West Nile, and Japanese encephalitis viruses [2,4]. The virion is spherical with an icosahedral symmetry and approximately 50 nm in diameter [2,3]. The C protein comprises the viral capsid which is surrounded by a lipid bilayer derived from the host, and the M and E proteins are anchored in the outer surface membrane. The 10.8 Kb ssRNA genome comprises a 5′ untranslated region (UTR), a single open reading frame, and a 3′ UTR. The open reading frame encodes a single polyprotein which is cleaved into the structural (C, prM and E) and nonstructural (NS1, NS2A, NS2B, NS3, NS4A, NS4B, and NS5) proteins [2,3]. ZIKV is an arthropod-borne virus (arbovirus) that is mainly transmitted to humans through the bite of mosquitoes [5,6], specifically *Aedes aegypti* and *Aedes albopictus* species [4]. ZIKV was first isolated from sentinel monkeys in Uganda in 1947 [7], and thereafter several ZIKV isolates were sampled from *Aedes* *africanus* mosquitoes [5]. Since the first reported human cases in 1952 [8], ZIKV has been sporadically detected in equatorial Africa and Asia over the next five decades [9,10]. Prior to 2007, only 14 human cases had ever been reported and ZIKV had been regarded as an arbovirus with mild clinical symptoms and inconsequential sequelae [11,12] that typically involved headache, fever, rash, conjunctivitis, arthralgia, and myalgia [2,11,13]. Fifty to 80% of infections remain asymptomatic [11,14,15]. Three genotypes of the virus have been identified: East African, West African, and Asian [13].

The first large outbreaks of ZIKV occurred in 2007 on the island of Yap in Micronesia, as the virus moved from Asia to the Pacific. The Yap outbreak is estimated to have affected ~73% of Yap residents older than three years of age [11,13]. The Yap outbreak was followed by a second large outbreak, this time in French Polynesia, during 2013–2014 [16,17]. During the French Polynesian outbreak, Guillain-Barré syndrome (GBS) was linked to ZIKV infection for the first time [18]. In May 2015, the World Health Organization (WHO) received the first reports of locally-transmitted ZIKV in Brazil [19]. In February 2016, due to the rapid expansion of ZIKV and a suspected causal relationship between the virus and microcephaly in Brazil, the WHO declared ZIKV a public health emergency of international concern. The epidemics in the Pacific and the Americas have seen increased rates of congenital neural abnormalities such as microcephaly, malformations of cortical development, brain calcifications, and hearing and vision loss [1,17,20,21,22], with infection often resulting in fetal demise during pregnancy [22,23]. Retrospective investigations of the 2013–2014 French Polynesian outbreak have linked microcephaly in newborns to ZIKV [17]. In adults, infection may lead to GBS [24,25], encephalitis [26], thrombocytopenia [27], and ocular and auditory disturbances [28]. To date, 84 countries have been affected, with almost one million cases, and at least 23 countries have reported a surge in the incidence of GBS (WHO, 10 March 2017).

## 2. Sexual Transmission of ZIKV

**Figure 1:**
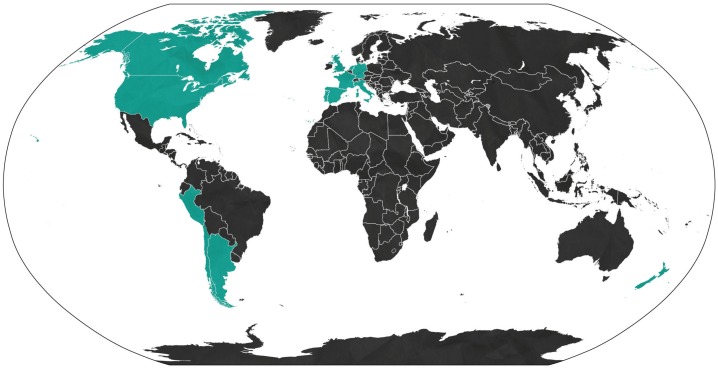
Countries outside of the endemic range of ZIKV that have reported cases of sexual transmission, 2011–2018 (shown in green).

Probable sexual transmission of ZIKV was first reported in 2011 when a scientist, who had contracted the virus while working in Senegal in 2008, infected his wife after returning home [29]. This was the first report of sexual transmission for any arbovirus to date. Since then, at least 14 countries outside the endemic range of ZIKV have reported person-to-person transmission of the virus (Figure 1). Both Asian and African genotypes of ZIKV have been reported to be sexually transmitted [29,30], suggesting that this mode of transmission appeared early in the evolution of the virus and prior to the divergence of genotypes [31]. Male-to-male [32], female-to-male [33], and male-to-female [29,30,34,35] cases of sexual transmission have been documented, with the latter being the most common [36]. Sexual transmission from men with no obvious symptoms has also been reported [37,38], although the prevalence is unclear since asymptomatic cases are inherently difficult to identify. Mathematical models predict the contribution of sexual transmission to the spread of ZIKV to be 3–4.8% [39,40]. However, one recent study suggests the risk of sustained sexual transmission may be much higher [41]. Differences in the age- and sex-specific attack rates of ZIKV have been observed, with women of childbearing age having the highest incidence of infection [36,42]. However, reporting bias may partially account for this pattern, as more women than men may have sought diagnosis due to increased fear of infection during pregnancy [39]. Although sexual transmission is unlikely to lead to sustained cycles of infection in areas without mosquito vectors, it could increase the likelihood of outbreaks occurring, and the size and duration of epidemics [39,40,41,43,44].

## 3. Persistent Shedding of ZIKV in Semen

ZIKV RNA has been detected in the semen of symptomatically [35,45,46,47,48,49] and asymptomatically-infected [50,51] men, sometimes for many months post onset of infection. Very high concentrations of ZIKV RNA can be found in semen during the clinically symptomatic phase of the infection [30]. One study has shown that up to 73% of infected men have detectable ZIKV RNA in their semen over the short term [48]. ZIKV has also been found attached to sperm [48,49,52,53], in particular to the mid-piece of mature spermatozoa [53], suggesting this could be a route of infection in addition to semen. The infectivity and longevity of ZIKV in semen varies [51]. The risk of sexual transmission by men is particularly high in the first few weeks of infection [54], with the median time between sexual contact and onset of symptoms in women estimated to be 9.5 days [43]. Persistent viral replication and shedding of infectious virus could, however, prolong this risk. Viral RNA and infectious virus have been detected in semen for up to 6 months [47] and 69 days [51,54] post-infection, respectively. Most studies have reported the presence of viral RNA in semen rather than infectious titers, possibly due to the difficulty of culturing viable virus from this fluid. How long infectious ZIKV persists in semen is therefore unclear. Nonetheless, the longevity of infectious ZIKV in semen, compared to vaginal fluids [55], indicates viral seeding and local replication occur in the genital organs and cells of the male reproductive tract (MRT). Persistently infected males may therefore be acting as potential reservoirs of ZIKV, which could account for some of the observed asymmetry in sexual transmission [36,41].

## 4. The MRT and Immune Privilege

**Figure 2:**
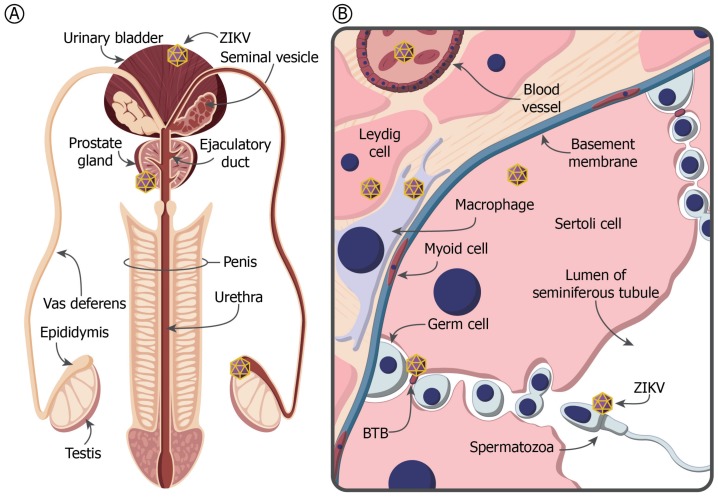
(**A**) Schematic representation of the male reproductive tract indicating potential ZIKV reservoirs. (**B**) Cross section of a portion of the seminiferous tubule within the testis. The seminiferous tubules contain the developing sperm cells and their supporting Sertoli cells. Sertoli cells form the lumen of the seminiferous tubules for release and transport of spermatozoa into the epididymis. Surrounding the seminiferous tubules are one or more continuous layers of peritubular myoid cells that function in the expulsion of spermatozoa out of the tubules and into the epididymis. The basement membranes of the seminiferous tubules are linked by tight junctions that, coupled with the myoid cells, form the blood-testis barrier (BTB). The interstitial compartment located between the tubules contains the Leydig cells, which are also essential for normal sperm development, maintenance of the blood-testis barrier, immune privilege, and Sertoli-germ cell junction assembly and disassembly.

Persistent viral replication and shedding of infectious virus from organs of the male reproductive tract (Figure 2A) could prolong the risk of sexual transmission [56]. Some regions of the MRT offer an immune-privileged environment that may lead to lowered fertility if disrupted by infection. Maintenance of immune privilege in the testis, the major organ where sperm are produced and androgens synthesized, is essential for healthy spermatogenesis. Within the testis, developing sperm are also protected from autoimmune attack by a physical blood-testis-barrier (BTB) formed by tight junctions between adjacent Sertoli cells that prevent immunoglobulin entry into the lumen (Figure 2B). An immune-privileged environment is also achieved through the suppression of normal immune responses that could lead to inflammation [57,58,59,60]. During male adolescence and throughout adult life, germ cells in the testes divide and differentiate to produce spermatogonia that are released into the lumen of the seminiferous tubules (Figure 2B). Immature sperm then travel to the epididymis and vas deferens where they mature and remain until ejaculation. Spermatozoa in the testes and regions of the epididymis are isolated from the host adaptive immune system to prevent the development of anti-sperm lymphocytes and, importantly, the production of anti-sperm antibodies (ASA). This immunosuppressive environment is enabled by the sequestration of antigens in phagocytosing Sertoli cells and testicular macrophages, downregulation of antigen presentation by macrophages and dendritic cells in the draining lymphatics, and the tight barrier formation between adjacent Sertoli cells preventing permeability of immunoglobulin [60]. Disruption of key cells involved in spermatogenesis, such as Sertoli and Leydig cells (Figure 2B), through infection and loss of immune privilege, could lead to autoimmune attack of spermatozoa and development of ASA, thereby lowering fertility.

## 5. ZIKV in the Testis and Prostate Gland

Prostatitis, hematospermia, and microhematospermia have been reported in ZIKV-infected men [29,34,61,62], as well as the presence of leukocytes in semen that is suggestive of inflammation in the MRT [62]. ZIKV may be breaching the BTB, disrupting immune privilege in the testes and replicating at these sites. ZIKV-infected human Sertoli cells show enhanced expression of cytokines and cell-adhesion molecules, increasing the adhesion of leukocytes and permeability of the BTB [63]. Inflammatory mediators released by ZIKV-infected testicular macrophages could also compromise the integrity of the BTB [63]. Low sperm counts have been observed in ZIKV-infected men [48,49,62], indicating that infection in the testis may be affecting sperm production. The lack of a correlation between the highest ZIKV loads in semen and serum [64] suggests that localized ZIKV replication occurs in the testicles and/or seminal glands [49]. The receptors used by ZIKV to enter the different cell types present in the MRT remain to be elucidated. However, the tyrosin kinase Axl is a major candidate entry receptor for ZIKV [65,66,67,68] and is expressed throughout the MRT, including the testes (particularly in Sertoli cells), the epididymis, and the prostate [69]. Axl is also an essential regulator in spermatogenesis [69]. Imaging of ZIKV-infected semen samples found that the virus colocalized to the Tyro3 receptor expressed at the mid-piece of mature spermatozoa, suggesting a role in ZIKV binding and entry [53]. Interestingly, Tyro3 receptors serve as entry ligands for Ebola and Marburg viruses [70], which have also been isolated from human semen and can be sexually transmitted [71]. Other as yet unidentified cell surface receptors may exist that could account for the tropism and sexual transmission of ZIKV.

The immunochemical detection of ZIKV inside the spermatozoa of a patient [49], as well as virus detection, isolation, and sexual transmission in the absence of spermatozoa [38,72,73], indicate that ZIKV could be present in semen as free virus particles or associated with cells. In the latter case, ZIKV could be transmitted to sperm by infected Sertoli cells, or virus particles could adsorb or penetrate spermatozoa during epididymal transit. The length of time required for sperm development in the seminiferous tubule (~2 months), relative to sperm maturation in the epididymis (~2 weeks), suggests most infectious virus could be acquired during the latter phase. However, additional studies are needed to determine the exact fate of ZIKV virions in the MRT. 

Virus may also be present in semen as a result of viral replication in the male accessory glands [71]. Sexual transmission of ZIKV from a vasectomized male to his female partner has recently been reported [72]. The presence of ZIKV in the semen of vasectomized men [72,73] has strongly implicated the prostate and seminal vesicles as potential reservoirs facilitating sexual transmission. Recently, in vitro infection of human prostate stromal, epithelial cells, and organoids demonstrated that ZIKV, but not dengue virus, actively infects and replicates in these cells, producing infectious virus in significant quantities [74]. The prostate is a strong candidate organ for prolonged viral shedding because it can host chronic infections with a variety of pathogens [56] and contributes a large proportion of seminal fluid during ejaculation [75].

## 6. Mouse Models of ZIKV in the MRT

Mice have proved the most tractable model to investigate ZIKV in the MRT, with a plethora of recent studies (Table 1) [31,52,76,77,78,79,80,81,82,83,84,85,86,87,88,89,90]. However, as ZIKV does not naturally replicate and cause disease in wild-type mice, studies of ZIKV pathogenesis have primarily utilized immunodeficient mice (Table 1). In such mouse models, the antiviral immune response is impaired, allowing replication and dissemination of ZIKV into different organs and tissues. Mouse models of sexual transmission have indicated the presence of infectious virus in 60–70% of ejaculates [31,79,84], and male-to-female sexual transmission in 50% of all matings [84]. Additionally, sexual transmission resulted in significantly greater morbidity and mortality and higher ZIKV titers in the female reproductive tract than subcutaneous or intravaginal inoculation [91]. A study using vasectomized mice showed that sexual transmission of ZIKV still occurred, despite semen containing significantly lower levels of infectious virus [84]. Overall, studies of pathogenesis in the MRT of mice have detected ZIKV in the testes [31,52,76,77,78,79,80,81,82,83,84,85,86,88,89,90] of all animals tested and the epididymis [31,52,76,77,78,80,84,86,87,88,89] of most mice (Table 1). ZIKV was also detected in the seminal fluid inside the lumen of the vas deferens [80] and the seminal vesicles [31,84,89] of some infected mice (Table 1). Although most studies did not investigate prostate tissues, one team reported negative results for ZIKV in the prostate [77], whereas two others did detect virus in this gland [88,89]. 

Androgen levels were altered in infected mice [79], concordant with ZIKV-induced reproductive hormone changes reported in men [48]. Inhibin B [76] and testosterone levels [76,77,78] were significantly decreased in mice, likely due to Leydig cell infection and apoptosis [76,78]. Furthermore, mouse ZIKV infection typically results in disruption of the BTB [77,86], breakdown of the epithelium and seminiferous tubules [76,77,79,86,87,88,90], inflammation and tissue injury to the epididymis and testis [76,77,80,83,84,87,89], and testicular atrophy [31,76,77,78,81,82,87]. Cytokine production within the testis, as well as infiltration of inflammatory cells, immune cells, and macrophages into this organ, seminiferous and epididymal tubules were observed [76,77,84,86,87,88]. ZIKV infection in mouse models also resulted in altered sperm morphology and motility, an absence of spermatozoa or reduction in total sperm counts, and a measurable reduction in fertility [52,76,77,78,79,81,87].

Testicular cells contribute much of the infectious virus shed in the seminal fluid of mice [84], however, mouse studies offer conflicting evidence regarding which exact cell types are targeted. In agreement with reports that human primary Sertoli cells support persistent ZIKV replication for at least six weeks [63,68], some mouse studies report Sertoli cells to be the major targets for ZIKV in testes [76,79,87,89,92]. Other studies report Leydig and myoid cells to be completely destroyed, resulting in the reduction in testosterone production and testicular atrophy in mice [77,78]. Virions attached to developing and mature sperm in the testes and epididymis, respectively, have been observed by transmission electron microscopy [52]. Some mouse models suggest that ZIKV infected cells are likely to be germinal spermatogonia or primary spermatocytes [76,77,83,84]. However, the detection of virus in epididymal spermatozoa 7 days post-infection strongly suggests that ZIKV directly infects spermatozoa in the epididymal lumen [76]. Sperm may therefore serve as a vehicle to transmit ZIKV in addition to semen. 

The observed difference in disease manifestation and severity between different mouse models could, in part, be explained by the use of varying mouse strains and ages at infection [89], ZIKV genotype, and virus dose and inoculation routes [76,85,86]. Although some of the key phenotypes observed in humans are recapitulated in immunodeficient mice, there are inherent limitations to using mouse models for the study of persistent ZIKV infection in the MRT. Compared to ZIKV-infected men [48,49], the injury to the MRT observed in mice is much more severe, and spermatogenesis more drastically affected [76,77]. Furthermore, the role of human immunity in ZIKV pathogenesis cannot be fully captured in immunodeficient mouse models. Using nonlethal mouse models [80,89] that allow for the long-term study of ZIKV infection kinetics and pathological progression, with an antibody response similar to macaques [85], could offer a way forward. 

## 7. Primate Models of ZIKV Pathogenesis in the MRT

Rhesus, cynomolgus, and pig-tailed macaques have been shown to be susceptible to a variety of ZIKV strains [93,94,95,96,97,98] and have been used to study ZIKV tropism and test ZIKV vaccine platforms [44,94,97,98,99]. Macaque models have been suggested as an alternative to mice because they develop clinical symptoms, viremia, widespread tissue infection, and a robust adaptive immune response comparable to human infection [93,95,98,100,101]. Clinical symptoms in infected macaques are generally mild [93,98,102], with plasma viremia peaking 2 to 6 days after infection and resolving within 10 to 14 days [93,95,97,102]. Infected rhesus macaques developed ZIKV-specific humoral and cell-mediated immune responses [93,94,95,102], protecting them from re-challenge with either homologous or heterologous ZIKV strains [97,102]. Both vector [93,95,96,97] and sexual [44] transmission routes have been studied in macaques. Asymmetry in ZIKV infectivity between males and females has also been observed in macaques [44]. Using in situ hybridization and quantitative reverse transcription PCR (RT-PCR) analysis to detect viral RNA, ZIKV dissemination into many tissues has been observed in macaques, including to the urogenital tract and shedding into mucosal secretions [94,95,98,100]. ZIKV persistence in the testes [95,100] and shedding of infectious virus in the semen [95] have been demonstrated. Importantly, the high viral load present in the testes of macaques, long after the systemic viral load has resolved [95,100], indicates that virus might be replicating at these anatomical sites. Immunohistochemistry of infected testes has shown virus localizing to Sertoli cells [95]. In addition, ZIKV has been detected in the seminal vesicles and prostate of rhesus and cynomolgus macaques for up to 35 days post infection [95,98]. However, not all studies using rhesus macaques have been able to detect ZIKV RNA in the testes [98], epididymis, and prostate [94]. Interestingly, pathogenesis studies have also detected ZIKV RNA in the kidney, bladder and urine [95,98], suggesting that ZIKV may also seed into semen from the urethra. The impact of ZIKV infection seems much less pronounced in immunocompetent macaque models versus mice. Although none of these macaque studies report the impact of ZIKV infection on testis structure and integrity and fertility, they clearly show that viral shedding continues unabated in the MRT.

## 8. Implications for the Development of Therapeutics and Vaccines

Reservoirs of persistent infection in the MRT could complicate the development of vaccines, antivirals, and/or other therapeutics for ZIKV. Proposed interventions and vaccines [103] need to be evaluated in their ability to clear persistent infection in the immune-privileged sites such as the male gonad. Evidence from HIV suggests that the testes may represent a distinctive virus sanctuary site in patients receiving suppressive antiviral therapy, with lingering virus detected in the testicles despite the virus been cleared from the bloodstream [104]. In this regard, although the antiviral Ribavirin was recently shown to suppress viremia in ZIKV-infected STAT1-deficient mice [105], it failed to suppress viral load in the brain, another immune-privileged site. Several compounds have shown promise as ZIKV prophylactic and therapeutic agents in vitro [66,68]. The antibiotic azithromycin has been shown to reduce ZIKV proliferation and cytopathic effects in vitro in glial cell lines, human astrocytes, and Sertoli cells [66,68]. Further studies are needed to investigate their effectiveness in vivo. Recently, the basic fibroblast growth factor (FGF2) was shown to be significantly upregulated in ZIKV-infected human Sertoli cells and to enhance viral replication and persistence [68]. Pre-treatment of Sertoli cells with either a neutralizing antibody to FGF2 or a FGF receptor inhibitor significantly inhibited ZIKV replication without affecting cell viability [68], thus indicating the therapeutic potential of FGF receptor antagonists.

A successful vaccine must provoke a subclass of immunoglobulin (IgG) inside the seminiferous tubules, as it has been proposed that only certain subclasses of IgG (i.e., IgG4) can cross the BTB [53]. Antibody treatments have shown promise in providing protection against persistent ZIKV infection. Human antibodies to the dengue virus E-dimer epitope (EDE1-B10), in addition to their inhibitory effects against dengue virus, have shown therapeutic potential against ZIKV [106]. EDE1-B10 treatment administered 1 to 3 days post infection was able to reduce viral persistence in the brain and testis, protect against ZIKV-induced inflammation, and damage to the seminiferous tubules, and preserve sperm counts [106]. The treatment, however, failed when administered 5 days after ZIKV infection. Polyclonal antibody treatment given 1 day prior to challenge [82], as well as live-attenuated and DNA-based vaccines [81,87], have protected mice against testicular atrophy and damage. In addition, DNA-based vaccines have been shown to induce sterilizing immunity against ZIKV challenge [99,107,108]. A vaccinia-based single vector construct, multi-pathogen vaccine, which encodes the structural polyprotein cassettes of both Zika and chikungunya (CHIKV) viruses, has recently been developed [90]. A single vaccination of *Ifnar1*^−/−^ mice induced neutralizing antibodies to both viruses and protected mice from CHIKV and ZIKV infection and disease, including testicular infection and pathology in males [90]. Vaccination resulted in complete clearance of ZIKV RNA in the testes from challenged male *Ifnar1*^−/−^ mice [90]. Initial murine studies have rapidly translated to clinical trials and have demonstrated that humans also develop neutralizing antibodies to the vaccine, which can provide passive immunity to mice during lethal ZIKV challenge [109]. Whilst a prophylactic ZIKV vaccine is achievable, the efficacy of current candidates as a therapeutic vaccine for chronically infected males remains unknown. The limited amount of immunoglobulin and lymphocytic infiltrate in the testes during infection may impede the success of current approaches.

## 9. Concluding Remarks and Future Prospects

Numerous questions regarding ZIKV infection in the MRT remain as yet unanswered (Box 1). The long-term effects of persistent ZIKV infection on male reproductive function, as well as on sperm production and fertility, including those exposed in utero, remain to be investigated. Of note, cryptorchidia, hypospadias, and micropenis have been described in newborns from infected mothers [110], but their prevalence is unknown. Although questions regarding pathogenesis can be answered using functional studies in animals, any effect of ZIKV infection on male fertility will only be detected with long-term epidemiological studies. Asymptomatic, persistent ZIKV replication in men and cryptic sexual transmission remain a risk to conception, given the large number of ZIKV infections that are silent. ZIKV-infected reproductive tissue (e.g., infected sperm) could pose a threat to patients seeking fertility services. Prospective studies of infected men are starting to reveal how long travelers from ZIKV-endemic areas should wait before trying to conceive naturally, donate gametes, or proceed with fertility treatments. Such data will aid in formulating appropriate public health guidelines to mitigate the risk of ZIKV infection through sexual transmission.

Box 1 **Key questions remaining to be answered regarding ZIKV in the MRT.**

- What are the cellular and molecular mechanisms of ZIKV persistence in the MRT?
- What is the origin of ZIKV in semen?
- What is the ZIKV entry receptor in the MRT?
- Which cells in the MRT are primarily infected following ZIKV attachment and entry?
- What are the viral and host characteristics that influence the infectivity and longevity of ZIKV in semen?

## Figures and Tables

**Table 1:** ZIKV localization in mouse models of MRT pathogenesis.

Mouse Genotype (Background).	ZIKV Genotype (Strain)	Inoculation Route	Testis (Infected Cells)	Epididymis	Seminal Vesicles	Vas Deferens	Prostate	Ref.
Wild Type (BALB/c) Dexamethasone Tx	Asian (PRVABC59)	IP	+ (ND)	+	ND	ND	+	[88]
Wild Type (C57BL/6) +	Asian (H/FP/2013)	SC	+ (SG, PS, ST, LC)	+	ND	ND	ND	[76]
anti-IFNαβR mAb	Afr (Dakar 41519)	SC	+ (SG, PS, ST, LC)	+	ND	ND	ND	[76]
*Rag1*^−/−^ (C57BL/6) + anti-IFNαβR mAb	Asian (Paraiba_01/2015)	IP	+ (SG, PS)	ND	ND	ND	ND	[83]
*Ifnar1*^−/−^ (C57BL/6)	Asian (ZIKV_SMGC-1)	IP	+ (LC, GC, PMC, SG)	+	−	ND	−	[77]
	Asian (Mex2-81)	SC	+ (LC)	+	ND	ND	ND	[78]
	Asian (PRVABC59)	SC	+ (ND)	ND	ND	ND	ND	[82]
	Asian (PRVABC59)	SC	+ (ST, MSC)	+	ND	ND	ND	[87]
	Asian (H/FP/2013)	SC	+ (ND)	ND	ND	ND	ND	[85]
	Asian (PRVABC59)	SC	+ (ST)	+	−	ND	+	[89]
	Asian (Mex2-81)	SC	+ (SG)	+	ND	ND	ND	[52]
	Asian (ZIKV_Natal_)	SC	+(ND)	ND	ND	ND	ND	[90]
(A129)	Asian (PRVABC59)	SC	+ (ND)	−	ND	ND	ND	[86]
	Asian (PRVABC59)	IP	+ (ND)	ND	ND	ND	ND	[81]
	African (MP1751)	SC	+ (ND)	+	ND	ND	ND	[86]
*Ifnar1*^−/−^ × *Ifngr*^−/−^ (AG129)	Asian (PRVABC59)	SC	+ (LC)	+	+	ND	+	[89]
	Asian (PRVABC59/FSS13025/P6-740)	SC	+ (ND)	+	+	ND	ND	[31]
	Asian (PRVABC59)	IP	+ (SG)	+	+	ND	ND	[84]
	African (Dakar 41524)	SC	+ (ND)	+	+	ND	ND	[31]
(AG6)	Asian (CAS-ZK01)	SC	+ (ST, MC)	ND	ND	ND	ND	[79]
*Irf3*^−/−^ × *Irf*7^−/−^ (C57BL/6)	African (MR766)	SC	+ (GC)	+	ND	+	ND	[80]

Abbreviations: Afr, African; mAb, monoclonal antibodies; SC, subcutaneous; IP, intraperitoneally; ND, not determined; SG, spermatogonia; PS, primary spermatocyte; ST, Sertoli cells; LC, Leydig cells; GC, germ cells; PMC, peritubular-myoid cells; MSC, maturing spermatogenic cells; MC, macrophage cells; + and −, detected and not detected, respectively. ZIKV Strains: PRVABC59 (Puerto Rico, 2015); H/FP/2013 (French Polynesia, 2013); Dakar 41519 (Senegal, 1984); Paraiba_01/2015 (Paraiba, 2015); ZIKV_SMGC-1 (Fiji and Samoa, 2016); Mex2-81 (Mexico, 2016); ZIKV_Natal_ (Brazil, 2015); MP1751 (Uganda, 1962); Dakar 41524 (Senegal, 1984); FSS13025 (Cambodia, 2010); P6-740 (Malaysia, 1966); CAS-ZK01 (Institute of Microbiology, Chinese Academy of Sciences, Beijing, China); MR766 (Uganda, 1947).

## Glossary

Arthralgia	Non-inflammatory joint pain.
Blood-testis-barrier	Physical barrier between blood vessels and the Sertoli cells of the seminiferous tubules in the mammalian testes.
Conjunctivitis	Inflammation of the outer layer of the eye and inside of the eyelid that causes the eye to turn pink.
Cryptorchidia	Condition in which one or both of the testes fail to descend from the abdomen into the scrotum.
Encephalitis	Inflammation of the brain.
Epididymis	Highly convoluted duct behind the testis, along which sperm passes to the vas deferens.
Guillain-Barré syndrome (GBS)	Autoimmune disease where antibodies and lymphocytes attack and damage the peripheral nerves causing weakness/paralysis and/or abnormal sensations and pain.
Hematospermia	Blood in the semen.
Hypospadias	A congenital condition in males in which the opening of the urethra is on the underside of the penis.
*Ifnar1*^−/−^ × *Ifngr*^−/−^ (AG129) mice	Interferon alpha, beta and gamma receptor deficient mice on a 129 background.
*Ifnar1*^−/−^ mice	Interferon alpha and beta receptor deficient mice.
Immune-privileged site	Sites that are able to tolerate the introduction of antigens without eliciting an inflammatory immune response. Immune-privileged sites include the central nervous system, the brain, the eye, and regions of the male reproductive tract.
*Irf3*^−/−^ × *Irf7*^−/−^ mice	Interferon 3 and 7 double knockout mice.
Leydig cells	Testosterone-producing cells located in the connective tissue surrounding the seminiferous tubules in the testicle.
Male accessory glands	In humans, these are the seminal vesicles, prostate gland, and the bulbourethral glands.
Microcephaly	Medical condition in which the brain does not develop properly resulting in a smaller than normal head.
Microhematospermia	Hematospermia not evident by macroscopic examinations of the semen, but detected by tests for occult blood.
Micropenis	An unusually small penis.
Myalgia	Pain in a muscle or group of muscles.
Organoids	Three-dimensional cell cultures that incorporate some of the key features of the represented organ.
Prostatitis	Inflammation of the prostate gland.
*Rag1*^−/−^ mice	Recombination activating gene 1 (Rag1) deficient mice.
Seminal glands	Accessory glands of the MRT, located between the bladder and the rectum that contribute approximately 60–70% of the ejaculate.
Seminiferous tubules	The site of the germination, maturation, and transportation of the sperm cells within the male testis.
Sertoli cells	Somatic cells of the testis that are part of a seminiferous tubule and facilitate the nourishment and progression of germ cells to spermatozoa.
Spermatocytes	Diploid cells formed through the process of spermatogenesis.
Spermatogenesis	The origin and development of the sperm cells within the male reproductive organs.
Spermatogonia	Undifferentiated male germ cell, formed in the wall of a seminiferous tubule and giving rise by mitosis to spermatocytes.
Spermatozoa	The mature motile male sex cell.
Testicular atrophy	Medical condition in which the testes diminish in size and may be accompanied by loss of function.
Testicular macrophages	Antigen-presenting cells, the most prevalent cell type in the testicular interstitium. They are in close morphological association and functional interaction with Leydig cells.
The male reproductive tract (MRT)	The male gonads, associated ducts and glands, and external genitalia that function during procreation.
Thrombocytopenia	Condition characterized by abnormally low levels of thrombocytes, also known as platelets, in the blood. This causes bleeding into the tissues, bruising, and slow blood clotting after injury.
Vas deferens	Tiny muscular tube in the MRT that carries sperm from the epididymis to the ejaculatory duct.
